# Maxillary First Molars with Six Canals Diagnosed with the Aid of Cone Beam Computed Tomography: A Report of Two Cases

**DOI:** 10.1155/2013/406923

**Published:** 2013-07-29

**Authors:** Mamta Kaushik, Neha Mehra

**Affiliations:** Department of Conservative Dentistry and Endodontics, Army College of Dental Sciences, Jai Jawahar Nagar, Secunderabad, Andhra Pradesh 500094, India

## Abstract

The case reports present the endodontic management of two maxillary first molars with six canals. The diagnosis of morphology of multiple canal systems was identified under magnification of the dental operating microscope and was confirmed with the help of cone beam computed tomography. This paper discusses the variations in the canal morphology and the use of the latest adjuncts in successfully diagnosing and treating unusual canal anatomy.

## 1. Introduction

A thorough knowledge of the root canal anatomy, its variations, the presence of additional roots, and unusual root canal morphology is essential, as it determines the successful outcome of endodontic treatment [1]. To ensure the long-term success of root canal treatment, it is essential to access, clean, and fill all of the canal spaces. However, the anatomic complexities and variations are constant challenges for successful endodontic therapy [2].

The morphology of the maxillary first molar has been extensively studied and reported in the literature. Traditionally the maxillary first molar exhibits three roots and three canals. The occurrence of a fourth canal ranges from 50.4% to 95% [3–7] and a fifth canal 2.25% [8], and a few authors have also reported cases with 6 canals [9, 10].

The occurrence of 2 canals in distobuccal root has been less frequent and has been reported in 3.6% of maxillary molars [4, 10, 11]. Palatine root canal variations were well established by Christie et al. [8, 12], who reported the endodontic treatment of maxillary molars with 2 palatine roots and classified these teeth as types I, II, and III, according to root degree of divergence. Others reported cases of maxillary first molar with two canals in each of the three roots [9, 10, 13–15].

The present cases report the successful management of maxillary first molars with three roots and six canals. The clinical findings were confirmed with the help of operating microscope and cone beam computed tomography (CBCT).

## 2. Case Report 1

A 43-year-old female patient presented with the chief complaint of pain in the left upper back tooth. The pain was continuous and aggravated on heat stimulation. The patient also complained of pain at night. The patient's medical history was noncontributory.

**Figure 1:**
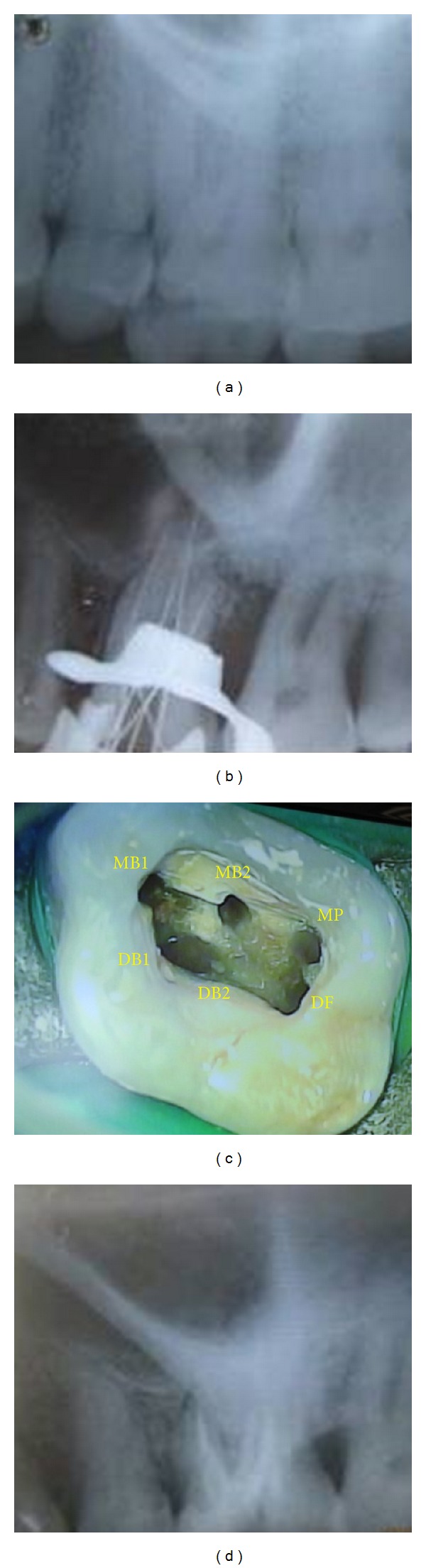
(a) Preoperative radiograph. (b) Working length radiograph showing multiple canals. (c) Access opening of the tooth showing multiple orifices. (d) Postobturation radiograph.

Clinical examination revealed the left maxillary first molar with a deep carious lesion which was tender on percussion. Electric pulp testing gave a premature response, indicative of inflammatory pulpal changes. The radiographic examination revealed a radiolucent lesion on the mesial aspect of the crown extending to the pulp (Figure 1(a)). After the clinical and radiographic examination, the left maxillary first molar was diagnosed with irreversible pulpitis and endodontic treatment was suggested to the patient.

The tooth was anaesthetised with 1.8 mL of 2% lidocaine containing 1 : 80,000 epinephrine (Lignox 2%, Indoco Remedies Ltd., Mumbai, India) followed by rubber dam isolation. A conventional endodontic access cavity was prepared. Clinical evaluation of the internal anatomy revealed 3 principal root canal systems: mesiobuccal (MB), distobuccal (DB), and palatal. After probing with a DG 16 endodontic explorer, small hemorrhagic points were noted 2 mm palatal to the MB and DB canals. As the dentin that was occluding the orifice of the palatal canal was removed, a second palatal canal was also identified. This was further evaluated and verified by a Surgical Operating Microscope (Seiler, St. Louis, MO). There seemed to be 2 distinct orifices in all the roots (Figure 1(c)). A sterile cotton pellet and an interim restoration of Cavit (3 M Espe, Seefeld, Germany) were placed in the pulp chamber to seal the access cavity.

To confirm this unusual morphology and to ascertain the pattern of the canals in a 3-dimensional manner, a cone beam computed tomography (CBCT) imaging of the tooth was advised. An informed consent was obtained from the patient, and a multislice CBCT scan of the maxillary left side was performed (Kodak 9000 3D) with a tube voltage of 80 KV and a tube current of 8 mA. The involved tooth was focused and a 3D morphology was obtained.

**Figure 2:**
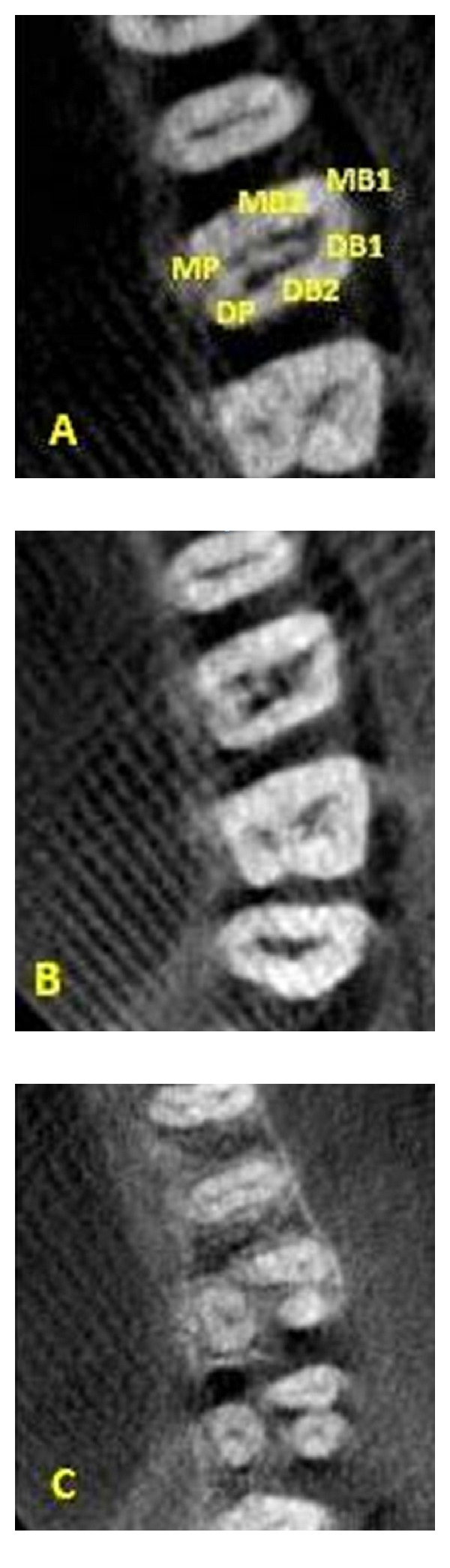
CBCT images showing 2 canals in each root.

The CBCT images confirmed the presence of six canals. The scans showed two mesiobuccal, two distobuccal, and two palatal canals (Figure 2). The mesiobuccal followed Vertucci's type IV classification. The distobuccal canals merged in the coronal third (2.4 mm from the orifice) and the palatal in the middle third (5.4 mm from the orifice) of the root to follow as a single canal (Vertucci's type II).

At the next visit, the working lengths of each canal were estimated by an electronic apex locator (Propex II, Dentsply) and confirmed with a radiograph (Figure 1(b)). The cleaning and shaping were performed using ProTaper nickel-titanium rotary instruments (Dentsply Maillefer, Switzerland). Irrigation between each instrument was done using 2.5% sodium hypochlorite solution and 17% EDTA. The canals were dried and obturation was performed using cold lateral compaction of gutta-percha (Dentsply Maillefer) and a resin-based sealer (AH Plus, Maillefer, Dentsply, Konstanz, Germany) (Figure 1(d)). The tooth was then restored with a posterior composite resin core (P60; 3 M Dental Products, St. Paul, MN). The patient was advised a full-coverage porcelain crown and was asymptomatic during the follow-up period.

## 3. Case Report 2

A 28-year-old female patient presented with the chief complaint of pain in the left upper back tooth. The pain was continuous and aggravated on heat stimulation. The patient's medical history was noncontributory. Based on clinical and radiographic examination a diagnosis of irreversible pulpitis was made and endodontic treatment was suggested to the patient.

**Figure 3:**
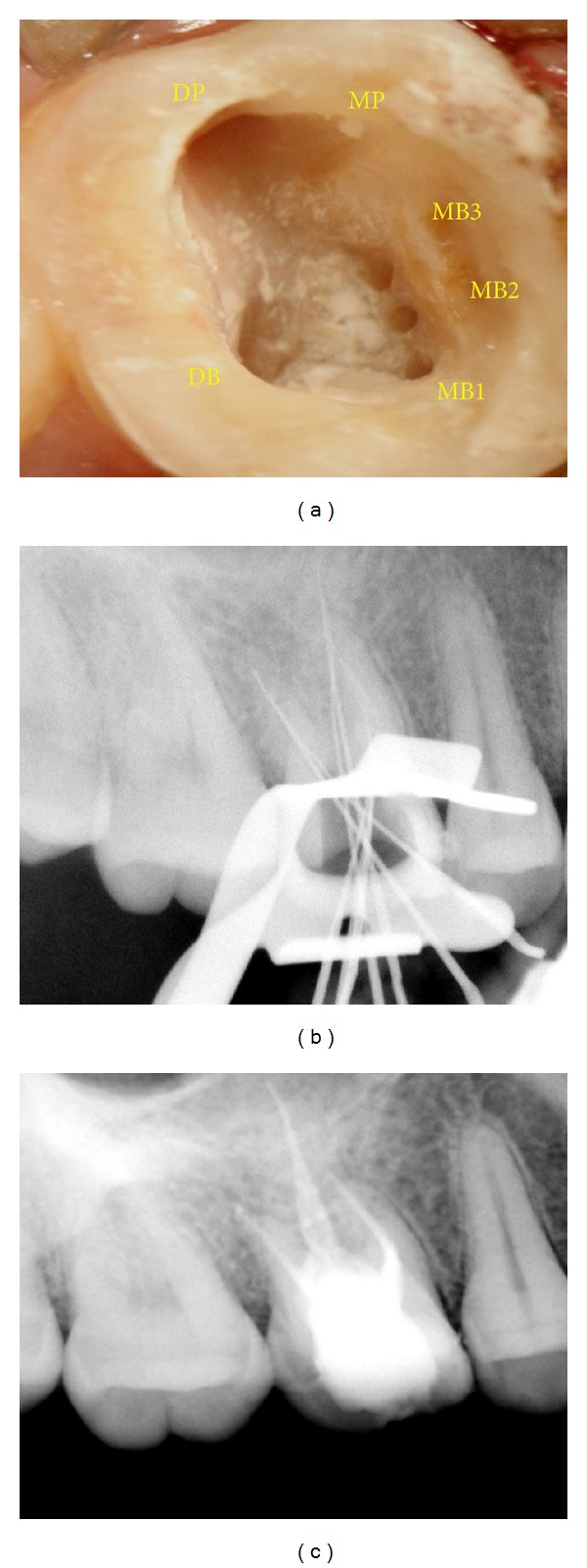
(a) Access opening showing three MB, two palatal, and one oblong distobuccal canal orifices. (b) Working length radiograph. (c) Postobturation radiograph.

The tooth was anaesthetised with 2% lidocaine containing 1 : 80,000 epinephrine (Lignox 2%, Indoco Remedies Ltd., Mumbai, India). A conventional endodontic access cavity was prepared under rubber dam isolation. Clinical evaluation of the internal anatomy revealed 3 principal root canal systems: mesiobuccal (MB), distobuccal (DB), and palatal. After probing with a DG 16 endodontic explorer, small hemorrhagic points were noted palatal to the mesiobuccal canal. On evaluation MB2 and MB3 were identified. Further exploration led to the identification of a second palatal canal. The distobuccal orifice also seemed to be indicating multiple canal system (Figure 3(a)). This was evaluated and verified by a Surgical Operating Microscope (Seiler, St. Louis, MO). The access cavity was sealed with Cavit (3 M Espe, Seefeld, Germany).

For further evaluation of this unusual morphology, a CBCT imaging of the tooth was advised. An informed consent was obtained from the patient, and a multislice CBCT scan of the maxillary left side was performed (Kodak 9000 3D) with a tube voltage of 80 KV and a tube current of 8 mA.

**Figure 4:**
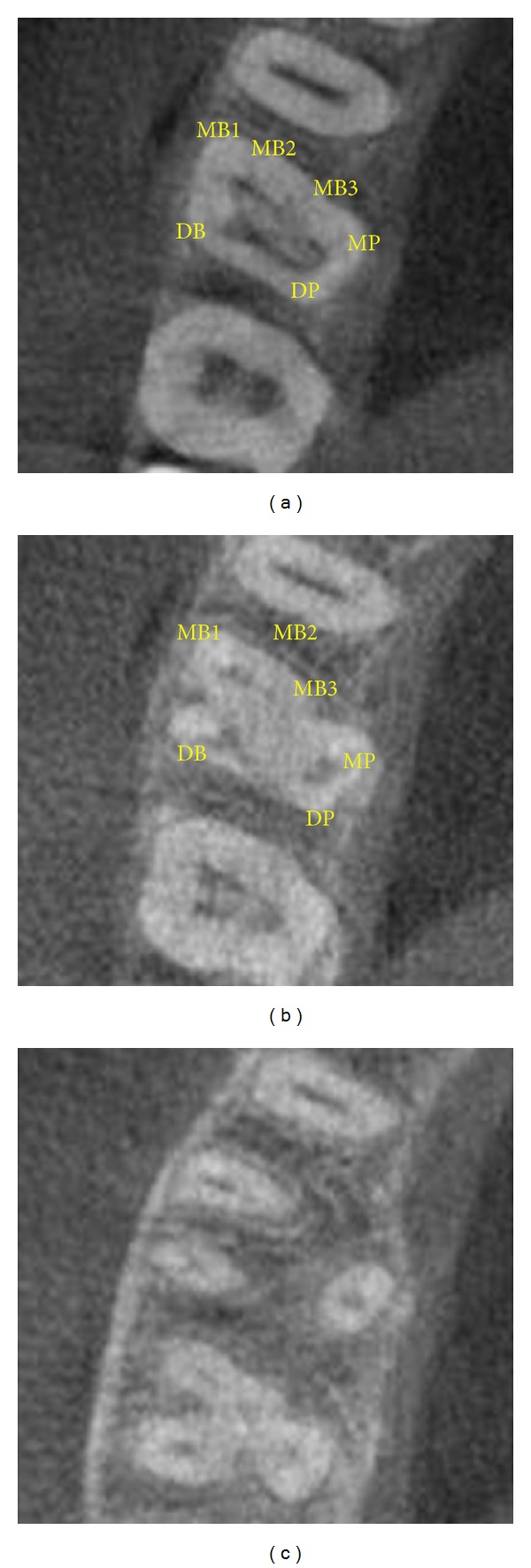
CBCT images showing distinct canal configuration.

The CBCT images confirmed the presence of six canals. The scans showed three mesiobuccal, two palatal, and an oblong distobuccal canal systems (Figure 4). The MB1 was an independent canal but the MB2 and MB3 merged (Vertucci's type II) to progress as one. The mesiopalatal and distopalatal canals (Vertucci's type II) merged in the middle third of the root to follow as a single canal.

At the next visit, the working lengths of each canal were estimated by an electronic apex locator (Propex II, Dentsply) and confirmed with a radiograph (Figure 3(b)). The cleaning and shaping were performed using ProTaper nickel-titanium rotary instruments (Dentsply Maillefer, Switzerland) with copious irrigation of 2.5% sodium hypochlorite solution and 17% EDTA. The canals were dried and obturation was performed using cold lateral compaction of gutta-percha (Dentsply Maillefer) and a resin-based sealer (AH Plus, Maillefer, Dentsply, Konstanz, Germany) (Figure 3(c)). The tooth was then restored with a posterior composite resin core (P60; 3 M Dental Products, St. Paul, MN). The patient was advised a full-coverage porcelain crown and was asymptomatic during the follow-up period.

## 4. Discussion

Anatomical aberrations are commonly observed in maxillary first molar ranging from one to seven canals [4]. It is generally accepted that maxillary first molar has three roots and three canals with a fourth canal (MB2) seen in 50.4–91% of cases [3–6]. The simultaneous occurrence of double canal system in all roots of a maxillary molar is an unusual finding [8, 9, 14, 15]. Case 1 highlights the unusual anatomy of maxillary first molar with double canals in all three roots.

Prevalence of additional root canals has been reported and discussed by several authors [15, 16]. Case 2 displayed a highly unusual morphology of 3 mesiobuccal, 2 palatal, and an oblong distobuccal canal systems.

Proper access opening and modifying the shape of the access to approach all orifices is a key to success in identifying and negotiating unusual anatomy of root canals [14]. In the present case reports, the conventional triangular access was modified to trapezoidal to improve access to the additional canals.

Diagnostic measures such as multiple preoperative radiographs, examination of the pulp floor with a sharp explorer, troughing of grooves with ultrasonic tips, staining the chamber floor with 1% methylene blue dye, performing the hypochlorite champagne bubble test, and visualising canal bleeding points are important aids in locating root orifices [17]. In the presented cases, examination of the pulpal floor to follow the dentinal map and exploration of haemorrhagic points with the DG16 was the first indication to hint at presence of extra orifices and canals.

An important aid for locating root canals is the Surgical Operating Microscope (SOM). It brings minute details into clear view by enhancing lighting and visibility. Studies have demonstrated that magnification and illumination by the SOM increased the identification of MB2 canals tremendously [3, 18–20]. The use of magnification aids in verifying the presence of morphologic variations.

Radiographic examination is an essential component for management of endodontic problems. But they produce only a 2D image of a 3D object resulting in superimposition of images [21]. CBCT is a valuable method for initial identification and effective evaluation of internal morphology of teeth [3, 22–25]. Although conventional CT scans produce a high level of detail, it is essential that the radiation dosage is kept as low as reasonably possible [26].

In the present cases, CBCT scanning was used for a better understanding of the complex root anatomy. For case 1, the images confirmed the presence of double canal system in three roots. The images showed that the palatal and distobuccal canals present with a Vertucci type II pattern and the mesiobuccal canals follow a Vertucci type IV configuration. Similarly, for case 2, the images confirmed the occurrence of multiple canal systems. The mesiobuccal root showed a Sert and Bayirli [27] type XV canal configuration. MB1 was an independent canal and MB2 and MB3 joined at the middle third to exit from one apical foramen. The palatal canals showed Vertucci's type II pattern.

The simultaneous occurrence of double canal systems in all roots of maxillary first molar is an unusual finding, as is the occurrence of 3 mesiobuccal and 2 palatal canals in the same tooth. Thus, it is important to be conscious to variations from the expected and to use all the armamentaria available to locate and treat the entire root canal system.

## 5. Conclusion

Although the incidence of root variations is rare, their importance should not be underestimated. Careful examination of radiographs and the internal anatomy of teeth are essential. The present cases confirm the necessity for meticulous examination of the pulpal floor at high magnification under sufficient illumination of the operating microscope and emphasize the importance of newer imaging techniques like CBCT in preoperative assessment.

## References

[1] Malagnino V, Gallottini L, Passariello P (1997). Some unusual clinical cases on root anatomy of permanent maxillary molars. *Journal of Endodontics*.

[2] Peters OA (2004). Current challenges and concepts in the preparation of root canal systems: a review. *Journal of Endodontics*.

[3] Baratto Filho F, Zaitter S, Haragushiku GA, de Campos EA, Abuabara A, Correr GM (2009). Analysis of the internal anatomy of maxillary first molars by using different methods. *Journal of Endodontics*.

[4] Cleghorn BM, Christie WH, Dong CCS (2006). Root and root canal morphology of the human permanent maxillary first molar: a literature review. *Journal of Endodontics*.

[5] Thomas RP, Moule AJ, Bryant R (1993). Root canal morphology of maxillary permanent first molar teeth at various ages. *International Endodontic Journal*.

[6] Kulid JC, Peters DD (1990). Incidence and configuration of canal systems in the mesiobuccal root of Maxillary first and second molars. *Journal of Endodontics*.

[7] Hartwell G, Appelstein CM, Lyons WW, Guzek ME (2007). The incidence of four canals in maxillary first molars—a clinical determination. *Journal of the American Dental Association*.

[8] Fogel HM, Peikoff MD, Christie WH (1994). Canal configuration in the mesiobuccal root of the maxillary first molar: a clinical study. *Journal of Endodontics*.

[9] Martínez-Berná A, Ruiz-Badanelli P (1983). Maxillary first molars with six canals. *Journal of Endodontics*.

[10] Bond JL, Hartwell G, Portell FR (1988). Maxillary first molar with six canals. *Journal of Endodontics*.

[11] Stone LH, Stroner WF (1981). Maxillary molars demonstrating more than one palatal root canal. *Oral Surgery Oral Medicine and Oral Pathology*.

[12] Christie WH, Peikoff MD, Fogel HM (1991). Maxillary molars with two palatal roots: a retrospective clinical study. *Journal of Endodontics*.

[13] Neaverth EJ, Kotler LM, Kaltenbach RF (1987). Clinical investigation (In Vivo) of endodontically treated maxillary first molars. *Journal of Endodontics*.

[14] Lee Y-Y, Yeh P-Y, Pai S-F, Yang S-F (2009). Maxillary first molar with six canals. *Journal of Dental Sciences*.

[15] Karthikeyan K, Mahalaxmi S (2010). New nomenclature for extra canals based on four reported cases of maxillary first molars with six canals. *Journal of Endodontics*.

[16] Kottoor J, Velmurugan N, Sudha R, Hemamalathi S (2010). Maxillary first molar with seven root canals diagnosed with cone-beam computed tomography scanning: a case report. *Journal of Endodontics*.

[17] Vertucci FJ (2005). Root canal morphology and its relationship to endodontic procedures. *Endodontic Topics*.

[18] Baldassari-Cruz LA, Lilly JP, Rivera EM (2002). The influence of dental operating microscope in locating the mesiolingual canal orifice. *Oral Surgery, Oral Medicine, Oral Pathology, Oral Radiology, and Endodontics*.

[19] Coelho De Carvalho MC, Zuolo ML (2000). Orifice locating with a microscope. *Journal of Endodontics*.

[20] Schwarze T, Baethge C, Stecher T, Geurtsen W (2002). Identification of second canals in the mesiobuccal root of maxillary first and second molars using magnifying loupes or an operating microscope. *Australian Endodontic Journal*.

[21] Patel S, Dawood A, Whaites E, Pitt Ford T (2009). New dimensions in endodontic imaging: part 1. Conventional and alternative radiographic systems. *International Endodontic Journal*.

[22] Nair MK, Nair UP (2007). Digital and advanced imaging in endodontics: a review. *Journal of Endodontics*.

[23] Patel S (2009). New dimensions in endodontic imaging: part 2. Cone beam computed tomography. *International Endodontic Journal*.

[24] Cotton TP, Geisler TM, Holden DT, Schwartz SA, Schindler WG (2007). Endodontic applications of cone-beam volumetric tomography. *Journal of Endodontics*.

[25] Tyndall DA, Rathore S (2008). Cone-beam CT diagnostic applications: caries, periodontal bone assessment, and endodontic applications. *Dental Clinics of North America*.

[26] Diederichs CG, Engelke WGH, Richter B, Hermann K-P, Oestmann JW (1996). Must radiation dose for CT of the maxilla and mandible be higher than that for conventional panoramic radiography?. *American Journal of Neuroradiology*.

[27] Sert S, Bayirli GS (2004). Evaluation of the root canal configurations of the mandibular and maxillary permanent teeth by gender in the Turkish population. *Journal of Endodontics*.

